# Extracellular proteasome-osteopontin circuit regulates cell migration with implications in multiple sclerosis

**DOI:** 10.1038/srep43718

**Published:** 2017-03-09

**Authors:** Chiara Dianzani, Elena Bellavista, Juliane Liepe, Claudia Verderio, Morena Martucci, Aurelia Santoro, Annalisa Chiocchetti, Casimiro Luca Gigliotti, Elena Boggio, Benedetta Ferrara, Loredana Riganti, Christin Keller, Katharina Janek, Agathe Niewienda, Chiara Fenoglio, Melissa Sorosina, Roberto Cantello, Peter M. Kloetzel, Michael P. H. Stumpf, Friedemann Paul, Klemens Ruprecht, Daniela Galimberti, Filippo Martinelli Boneschi, Cristoforo Comi, Umberto Dianzani, Michele Mishto

**Affiliations:** 1 Department of Drug Science and Technology, University of Turin, 10126 Torino, Italy; 2 Department of Experimental, Diagnostic and Specialty Medicine (DIMES), University of Bologna, 40126 Bologna, Italy; 3 Centre for Integrative Systems Biology and Bioinformatics, Department of Life Sciences, Imperial College London, SW7 2AZ London, UK; 4 Institute of Neuroscience, Centro Nazionale delle Ricerche (CNR), 20129 Milano, Italy; 5 IRCCS Humanitas, 20089 Rozzano, Italy; 6 Interdisciplinary Research Centre of Autoimmune Diseases (IRCAD), University of Piemonte Orientale, Amedeo Avogadro, 28100 Novara, Italy; 7 Institut für Biochemie, Charité - Universitätsmedizin Berlin, 10117 Berlin, Germany; 8 Berlin Institute of Health, 10117 Berlin, Germany; 9 Neurology Unit, Department of Pathophysiology and Transplantation, University of Milan, “Dino Ferrari” Centre, Fondazione IRCCS Cà Granda, Ospedale Maggiore Policlinico, 20100 Milano, Italy; 10 Department of Neuro-rehabilitation and Laboratory of genetics of Neurological complex disorders, Institute of Experimental Neurology (INSPE) Scientific Institute San Raffaele, 20132 Milano, Italy; 11 Department of Translational Medicine, Section of Neurology, University of Piemonte Orientale, 28100 Novara, Italy; 12 AG Klinische Neuroimmunologie des Neurocure Clinical Research Center, Charité - Universitätsmedizin Berlin, 10117 Berlin, Germany; 13 Clinical and Experimental Multiple Sclerosis Research Centre - Universitätsmedizin Berlin, 10117 Berlin, Germany; 14 Experimental and Clinical Research Center, Max Delbrueck Center for Molecular Medicine and Charité - Universitaetsmedizin Berlin, Berlin, Germany; 15 Department of Neurology, Charité - Universitätsmedizin Berlin, 10117 Berlin, Germany; 16 Department of Health Science, University of Piemonte Orientale, 28100 Novara, Italy

## Abstract

Osteopontin is a pleiotropic cytokine that is involved in several diseases including multiple sclerosis. Secreted osteopontin is cleaved by few known proteases, modulating its pro-inflammatory activities. Here we show by *in vitro* experiments that secreted osteopontin can be processed by extracellular proteasomes, thereby producing fragments with novel chemotactic activity. Furthermore, osteopontin reduces the release of proteasomes in the extracellular space. The latter phenomenon seems to occur *in vivo* in multiple sclerosis, where it reflects the remission/relapse alternation. The extracellular proteasome-mediated inflammatory pathway may represent a general mechanism to control inflammation in inflammatory diseases.

Osteopontin (OPN), a component of bone matrix and a soluble pleiotropic cytokine, plays a pivotal role in several diseases such as tumors, myocardial and kidney dysfunctions, and autoimmune diseases. OPN has a particular relevance in multiple sclerosis (MS), a disease in which the autoimmune response targets the myelin sheaths of the central nervous system (CNS)^1^. Indeed, in MS secreted OPN stimulates the expression of Th1 and Th17 cytokines, inhibits apoptosis of autoreactive T cells, and regulates leukocyte adhesion, migration, and trafficking into the CNS by binding to CD44 and various integrins^2^. Increased concentrations of OPN occur in peripheral blood and cerebrospinal fluid during the relapse phase in MS patients^3^,^4^,^5^. Moreover, OPN is strongly expressed in MS lesions. A similar phenomenon occurs also in the experimental autoimmune encephalomyelitis (EAE), an MS mouse model in which the administration of the OPN results in rapid induction of relapse, increased level of neurological defects and progression of the disease^6^.

Secreted OPN molecules are cleaved by matrix metalloproteinases and thrombin^1^. Thrombin cleaves the full length OPN (OPN-FL) into N-terminal (OPN-N) and C-terminal (OPN-C) fragments, which exert different biological activities^1^,^7^,^8^. Another protease present in different types of biological fluids, including blood, is the 20S proteasome. Depending on its incorporated catalytic subunit pattern proteasome is named as either standard proteasome, intermediate-type proteasome or immunoproteasome. Standard proteasome is the most common proteasome isoform, whereas intermediate-type proteasome and immunoproteasome are expressed during inflammation or in immune cells^9^. The proteasome isoforms possess different proteolytic dynamics^10^,^11^,^12^. The difference in protein turnover results in preferential tasks carried out by either standard proteasome or immunoproteasome. For instance, immunoproteasome specifically regulates some aspects of the cytokine-mediated inflammation and cell-mediated immunity^9^. It is generally believed that these immunological tasks are carried out by the proteasome only as intracellular protease; indeed, the role of extracellular proteasomes as well as their cellular origin and the mechanisms of release remain elusive^13^. However, it is known that extracellular proteasome purified from plasma is proteolytically active^14^. Active release rather than passive leakage from injured/apoptotic cells was suggested to cause the early increase of extracellular proteasome levels in sepsis and severe injury^15^. Active mechanisms of proteasome release have been proposed through various types of extracellular vesicles (EVs), *i.e.* microparticle/ectosomes shedding from the surface of T lymphocytes^16^, or exosomes derived from endocytic compartments of mesenchymal stem cells^17^. In patients with solid tumors and hematological malignancies, extracellular proteasome levels increase and correlate with occurrence, severity, as well as clinical outcome of the diseases^18^,^19^,^20^. In some autoimmune diseases, such as rheumatoid arthritis, MS and systemic lupus erythematosus, extracellular proteasome levels are increased and are markers of cell damage and immunological activity^21^,^22^.

No function of proteasome in the extracellular space has been demonstrated so far.

## Results and Discussion

### 20S proteasome processes OPN molecules and modulates their chemotactic activities

**Figure 1:**
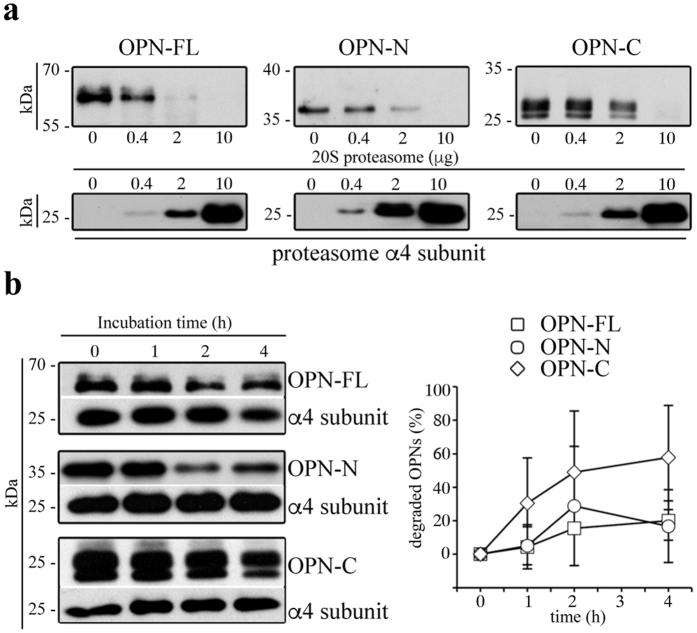
Extracellular 20S standard proteasomes cleave OPN molecules. (**a**) Representative *in vitro* digestion (n = 3) of recombinant OPN-FL, OPN-N, OPN-C by different amount of human erythrocyte 20S standard proteasome. (**b**) Degradation kinetics of OPN-FL, OPN-N and OPN-C by T2 20S standard proteasomes are shown by representative Western Blot assay (left panel; the proteasome α4 subunit is used as control marker since it is incorporated in each proteasome). The density of the Western Blot bands is measured and the corresponding relative OPNs’ degradation computed (right panel; mean and SD of 4–5 independent experiments measured in duplicate). The relevant bands of the Western Blot assays shown here are cropped from the full blots shown in Supplementary Fig. 1. No significant differences between the degradation rate of the three OPNs are observed by Kruskal-Wallis test. In (**b**) we use a ratio OPNs/proteasome that roughly resembles that observed in the peripheral blood of healthy and MS donors (Fig. 5) and that used in the cell migration experiments (Fig. 2).

To investigate whether extracellular proteasome could be involved in the OPN-mediated inflammatory events, we first investigate whether 20S proteasome can fragment OPN molecules in an ubiquitin-independent manner. We use purified 20S proteasome because there is no evidence that the entire ubiquitin-proteasome system is present and functional in the extracellular space. Furthermore, 20S proteasome is the only active proteasome form that has been identified in extracellular microparticles^16^. Therefore, we perform *in vitro* degradation of different portions of recombinant OPNs, *i.e.* OPN-FL (OPN-FL_17–314_-6His), OPN-N (OPN-FL_17-168_-6His) and OPN-C (OPN-FL_169-314_-6His) by purified erythrocyte 20S standard proteasomes. Testing different ratios of proteasome/OPNs, we observe a concentration-dependent degradation of OPN substrates, thereby showing that 20S standard proteasome can cleave OPNs outside the cell in an ubiquitin-independent manner (Fig. 1a and Supplementary Fig. 1). Furthermore, we investigate the degradation rate of the three OPNs by using 20S standard proteasome derived from a different cell source, *i.e.* derived from T2 cells. Thereby we verify that the OPN processing is not a singularity of erythrocyte 20S standard proteasome (Fig. 1b and Supplementary Fig. 1).

Since a key function of OPNs during inflammation is the regulation of cell chemotaxis, we study whether proteasome-mediated processing of OPNs affects the *in vitro* chemotaxis of different cell types, *i.e.* human umbilical vein endothelial cells (HUVEC), peripheral blood lymphocytes (PBLs) and monocytes (PBMs). These cells are recruited through OPN-mediated chemotaxis to the inflammation site in different physiological and pathological conditions. HUVECs are a standardized model for investigating the behavior of vascular endothelial cells, which are key players in inflammation by regulating inflammatory cell trafficking. Furthermore, the migration of PBLs and PBMs in the CNS is considered to be an important pathological inflammatory factor for the MS development.

**Figure 2:**
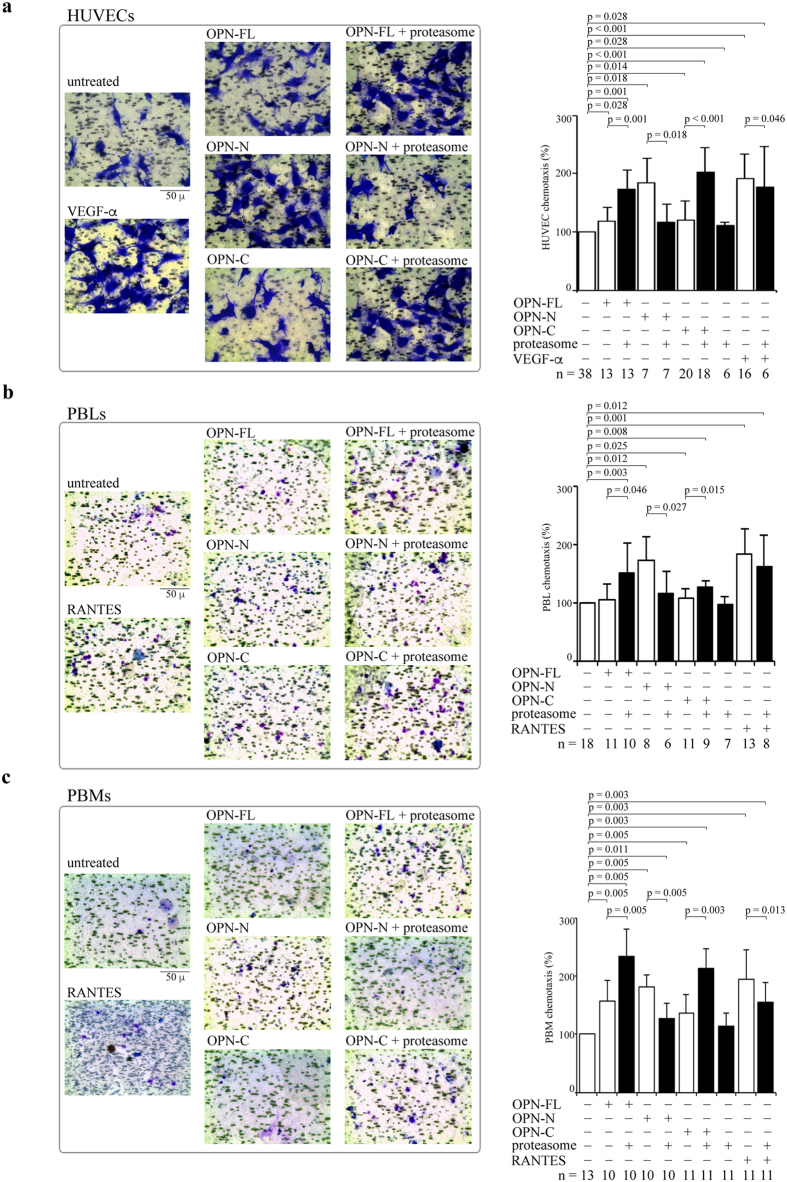
Extracellular proteasome modulates chemotactic activity of OPN molecules. (**a**) Representative pictures of migrated HUVECs (stained by crystal violet) treated with OPNs ± 20S proteasome are displayed. The quantitative effect of OPNs ± 20S proteasome (and VEGF-α as positive control) on the HUVEC chemotaxis (n = 6–38) is shown. (**b**) Representative pictures of migrated PBLs treated with OPNs ± 20S proteasome are displayed. The quantitative effect of OPNs ± 20S proteasome (and RANTES as positive control) on the chemotaxis of PBLs (n = 6–18) is shown. (**c**) Representative pictures of migrated PBMs treated with OPNs ± 20S proteasome are displayed. The quantitative effect of OPNs ± 20S proteasome (and RANTES as positive control) on the chemotaxis of PBMs (n = 11–13) is shown. In (**a–c**) cell migration is measured by applying the Boyden chamber migration assay; values are percentage of treated *vs* untreated cells that migrated after 20 h and are the mean and the SD of independent experiments. Wilcoxon test for paired samples *p* < 0.05 are shown.

HUVECs, PBLs and PBMs are treated with OPNs pre-incubated or not with 20S proteasomes. The chemotaxis is measured in a Boyden chamber migration assay after 20 h (Fig. 2a). We use as positive control the well-known chemotactic factors VEGF-α for the HUVECs and RANTES for PBLs and PBMs.

In HUVECs, the presence of 20S proteasome significantly hampers the chemotactic stimulus by OPN-N whereas OPN-FL and OPN-C gain chemotactic activity upon proteasome digestion. Proteasome has only marginal effects *per se* on spontaneous migration or in the chemotaxis induced by VEGF-α (Fig. 2a). 20S proteasome also significantly hampers the chemotaxis of PBLs induced by OPN-N, whereas it boosts the chemotaxis upon processing of OPN-FL and OPN-C. The proteasome has neither effects *per se* on chemotaxis nor on the RANTES-induced chemotaxis (Fig. 2b). Similar effects of proteasome-catalyzed OPN cleavage are observed in PBMs; indeed, proteasome-mediated digestion of OPN-N significantly reduces its chemotactic activity, whereas it significantly enhances the PBMs’ chemotaxis induced by OPN-FL and OPN-C. Proteasome has no effect in absence of OPNs but it reduces, mildly although significantly, the chemotactic activity of RANTES in PBMs (Fig. 2c).

### Specific proteasome-generated fragments of the OPN-C stimulate HUVEC and PBL chemotaxis

The fact that 20S proteasome can cleave OPN-FL and OPN-C thereby increasing their chemotactic activity suggests that 20S proteasome-catalyzed degradation of OPN-FL and OPN-C might unmask cryptic OPN chemotactic fragments. Such fragments would then be able to activate chemotaxis better than when they were included and folded in the OPN-FL. Similar mechanism has been proposed for the activation of other cryptic sites of OPN-FL upon processing by thrombin^2^,^7^.

**Figure 3:**
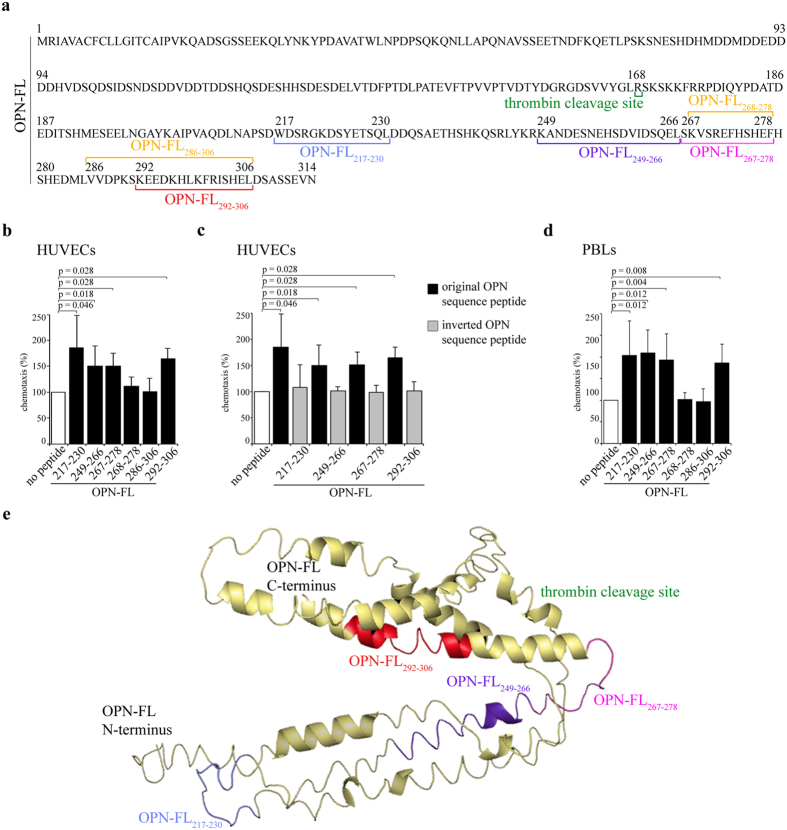
Location and chemotactic effect of proteasome-generated OPN fragments towards HUVECs and PBLs. **(a**) The sequence of the OPN-FL (P10451.1) and of the studied proteasome-generated OPN fragments is here disclosed. (**b**) The effect of different OPN fragments (100 nM) on the HUVEC chemotaxis is shown.(**c**) The comparison between the efficacy of the proteasome-generated OPN fragments and of the corresponding peptides with inverted sequence in inducing chemotaxis in HUVECs is shown. (**d**) The effect of the studied OPN fragments (50 nM) on the PBL chemotaxis is shown. (**b–d**) Values are reported as percentage of treated *vs* untreated cells that have migrated after 20 h and are expressed as the mean and the SD of independent experiments (n = 4–14). Cell migration is measured by the Boyden chamber migration assay. Wilcoxon test for paired samples *p* < 0.05 are shown. (**e**) Tertiary structure prediction of OPN-FL. The predicted structure is shown as a cartoon representation indicating disordered regions and α-helices. Highlighted are the sequences produced by proteasome that show strong chemotactic activity and the thrombin cleavage site; colors correspond to (**a**). The structure is predicted with I-TASSER server.

To identify these active fragments, we digest recombinant OPN-C by 20S proteasome and analyze the products by mass spectrometry. Among more than hundred identified fragments (data not shown), we select 6 fragments, *i.e.* OPN-FL_217-230_, OPN-FL_249-266_, OPN-FL_267-278_, OPN-FL_268-278_, OPN-FL_286-306_ and OPN-FL_292-306_ (Fig. 3a), showing dominant mass spectrometry ion peak areas in a 20 h degradation of OPN-C by 20S proteasome from erythrocytes (Supplementary Fig. 2). These peptides are synthetized and analyzed for their ability to induce HUVEC chemotaxis. Among them, four peptides – *i.e.* OPN-FL_217-230_, OPN-FL_249-266_, OPN-FL_267-278_ and OPN-FL_292-306_ - exert their chemotactic activity towards HUVECs in a dose-dependent manner (Supplementary Fig. 3). In particular, they are effective at the dose of 100 nM (Fig. 3b), which is likely the maximal OPN fragment concentration generated by extracellular 20S proteasome during our chemotaxis experiments (Fig. 2). Their activity is sequence-dependent, and not only related to chemical properties of the peptides, since control peptides with inverted sequences compared to the functional peptides have no chemotactic activity towards HUVECs (Fig. 3c).

The same response towards the 6 OPN-derived peptides is observed for the cell migration of PBLs. Indeed, the four peptides OPN-FL_217-230_, OPN-FL_249-266_, OPN-FL_267-278_ and OPN-FL_292-306_ are chemoattractant also towards PBLs, whereas the peptides OPN-FL_268-278_, OPN-FL_286-306_ exert no effect (Fig. 3d).

According to the prediction of the OPN-FL 3D structure (Fig. 3e) the four active OPN fragments are either buried in the OPN-FL structure, or part of loops and strongly secondary structured alpha-helices.

### OPNs reduce the release of proteasome by endothelial cells in the extracellular space

**Figure 4:**
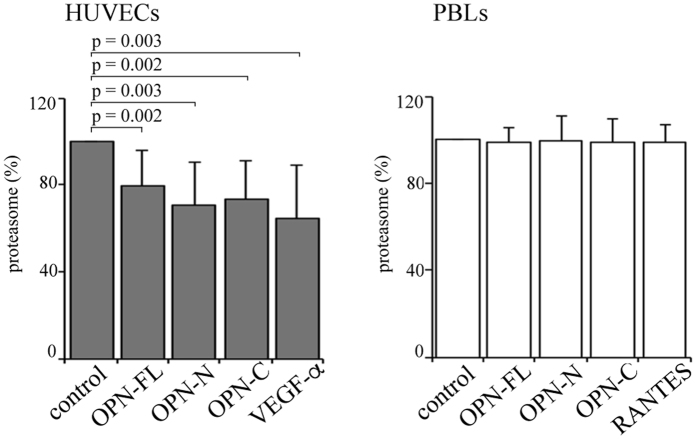
OPNs and VEGF-α partially inhibit the release of proteasome by HUVECs. The proteasome release by HUVECs (left panel) and PBLs (right panel) in serum-free medium with OPN-FL, OPN-N, OPN-C and VEGF-α (with HUVECs) or RANTES (with PBLs) is shown. Values are reported as proteasome concentration ratio of treated *vs* untreated cells and they correspond to the mean and the SD of independent experiments (HUVECs, n = 14; PBLs, n = 10) measured by ELISA. Statistically significant variations from the control are detected by Wilcoxon test for paired samples and *p* < 0.05 are annotated.

In biological systems, negative feedback loops exist to control the activity of mediators. Thus, we investigate whether a reciprocal regulation could exist between OPN and extracellular proteasome, whereby OPNs regulate proteasome release in the extracellular space. To this end, we measure the concentration of extracellular proteasome in cultures of HUVECs, PBLs or PBMs upon treatment with the three OPNs, and either VEGF-α or RANTES, respectively. We observe a significant decrease in the extracellular proteasome concentration in the medium of HUVEC cultures upon stimulation with either each of the three OPNs or with VEGF-α compared to untreated cells (Fig. 4). On the contrary, these cytokines do not have any effect on proteasome released by PBLs (Fig. 4); a similar lack of effect is also detected with PBMs (data not shown).

### An inverse correlation between extracellular OPN and proteasome is confirmed in peripheral blood of RRMS patients

We then verify whether this negative feedback loop between extracellular OPN and proteasome concentration is detectable also *in vivo*. This could be detected through an inverse correlation of extracellular OPN and proteasome concentration. We would expect that the proteasome release is regulated by different factors in non-inflamed and inflamed subjects. In the latter, when OPN function and concentration raises, the impact of OPNs in the release of proteasome might become evident and an inverse correlation between OPN and proteasome concentration should be detectable in bodily fluids. This should be the case in the relapsing-remitting multiple sclerosis (RRMS), which is the most prevalent form of MS, and it is anticipated in 85% of people by an acute onset named clinically isolated syndrome (CIS). RRMS is characterized by disability episodes (relapses) followed by a complete or partial recovery (remission). During (or right before) the relapse of RRMS patients, the levels of OPN rise together with the exacerbation of the CNS inflammation, while they decrease in the following remission^1^.

**Figure 5:**
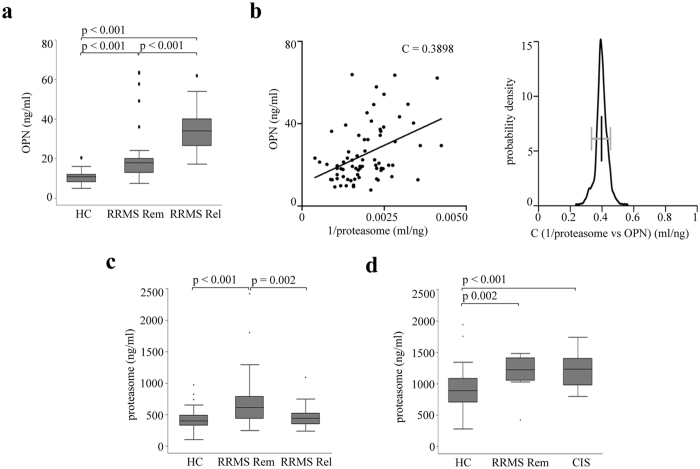
Serum OPN and proteasome levels inversely correlate in RRMS patients. **(a)** Serum OPN concentration is significantly increased in an Italian cohort of RRMS patients in remission (RRMS Rem, n = 48) and relapse (RRMS Rel, n = 24) compared to healthy controls (HC; n = 28), as well as in RRMS Rel *vs* RRMS Rem. The Kruskal-Wallis test is applied with a Bonferroni correction for multiple comparisons, and *p* < 0.05 are reported. (**b**) Serum OPN and proteasome concentration in Italian RRMS patient cohort (n = 72) are nonlinearly and inversely correlated as shown by plotting OPN *vs* 1/extracellular proteasome concentrations (Pearson’s test, *p* < 0.001; C value is shown in the chart). Bootstrap test using 1000 samples with 100% of the data shows a significant correlation coefficient between 0.25 and 0.55 (right panel). (**c)** Serum proteasome concentration in Italian healthy donors (n = 62), RRMS patients in remission (RRMS Rem, n = 50) or in relapse (RRMS Rel, n = 25). (**d)** Serum proteasome concentration in German healthy controls (n = 50), RRMS patients in remission (RRMS Rem, n = 12) and CIS patients (n = 35). 3–6 technical ELISA replicates for each samples are used in the analysis. Kruskal-Wallis test is applied for multiple comparison with Bonferroni correction and *p* < 0.05 are reported. In (**a,c,d**) the median, the 25–75 quartiles (the grey box), the 0–100 quartiles (the error bars) and the outliers are shown.

In line with our hypothesis, in the sera of a first Italian cohort (Supplementary table 1), we observe a significantly higher concentration of OPNs in RRMS patients in relapse compared to those in remission and healthy donors (Fig. 5a), and a significant inverse non-linear correlation between extracellular proteasome and OPN concentrations by pooling together relapses and remissions (Fig. 5b). To further assess the robustness of this correlation, we perform a bootstrap analysis to obtain confidence intervals. All bootstrap samples show a significant correlation coefficient between 0.25 and 0.55, which assures that the observed correlation is stable to potential outliers (Fig. 5b). This inverse correlation within the RRMS cohort suggests that the dramatic increase of OPN levels in the relapse leads to a corresponding decrease of serum proteasome concentration. According to our hypothesis, such effect should disappear during the remission when the secreted OPN concentration drops. This is indeed what we observe in the serum of the RRMS cohort: the extracellular proteasome levels are significantly higher in Italian RRMS patients in remission compared to those in relapse (Fig. 5c). They are also higher than those measured in healthy controls in agreement with the study of Minagar and colleagues^22^. This latter observation is confirmed in a second German cohort of RRMS patients in remission compared to healthy donors as well as of CIS patients compared to healthy donors (Fig. 5d).

### EVs-free extracellular proteasome varies amongst disease groups

Extracellular vesicles (EVs) have been hypothesized to be one of the mechanisms for the proteasome release in the extracellular space. EVs have been also suggested as the active delivery mechanism of biological mediators in peripheral blood and other bodily fluids of several diseases including MS^16^,^23^,^24^. Hence, we investigate whether serum EVs might be responsible for the differences in extracellular proteasome concentration we observed in RRMS and CIS cohorts compared to healthy donor cohort.

**Figure 6:**
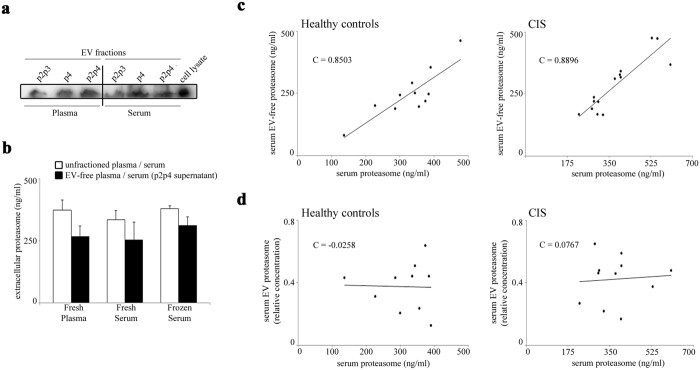
The main portion of serum extracellular proteasome is not stored in isolated EVs. **(a)** Representative Western blot analysis (n = 3) of p2p3 (large vesicles), p4 (small vesicles) and p2p4 (all vesicles) protein extracts are shown. They derive from healthy donor’s freshly-isolated plasma and serum samples. Cell lysate from lymphoblastoid cell lines is used as control. (**b)** The quantitative evaluation of extracellular proteasome concentration is here shown and is obtained by applying ELISA on the fresh plasma (n = 5) and serum (n = 3) or frozen serum (n = 3) of healthy donors, or on their corresponding EV-free supernatants, which are obtained by removing the p2p4 vesicles upon ultra-centrifugations. (**c)** The correlation between the proteasome concentration in the total serum and the EV-free (p2p4-free) serum supernatant of a subset of German healthy donor (n = 11) and CIS patient (n = 14) cohorts (measured by ELISA) is shown. A statistically significant positive correlation is observed between the extracellular proteasome concentration in the EV-free (p2p4-free) serum supernatant and in the unfractioned serum (Pearson’s test; in healthy donors, *p* = 0.001; in CIS patients, *p* < 0.001). (**d)** The proteasome concentration in p2p4 vesicles of a subset of healthy donors (n = 10) and CIS patients (n = 11) is shown. It is measured by Western blotting and by using anti-α6 proteasome subunit antibody. It is expressed as proteasome concentration relative to that of the cell lysate of lymphoblastoid cell lines. No correlation between the extracellular proteasome concentration measured in serum p2p4 vesicles or in whole serum is observed. Pearson ’s test C values are reported in each chart.

Different factors might affect EV stability in extracellular fluids and lead to difference when plasma and serum are considered^25^. Therefore, we initially analyze the proteasome content in large vesicles (p2p3, >100 nm), small vesicle/exosomes (p4 < 100 nm) or all vesicles (p2p4), as well as the corresponding supernatants centrifuged from freshly isolated healthy donor plasma and serum by western blotting. Proteasomes are present in both large and small vesicle fractions and at a similar extent comparing plasma and serum samples (Fig. 6a). By ELISA on healthy donor plasma and serum supernatants, we find that a large portion of extracellular proteasomes is not associated with EVs, both in frozen and fresh samples (Fig. 6b). Accordingly, extracellular proteasomes are mainly present in the EVs-depleted serum in a sub-cohort of both German healthy donors (n = 11; mean = 74.4 ± 14.8%) and German CIS patients (n = 14; mean = 74.9 ± 12.0%). In the same sub-cohorts, the amount of extracellular proteasomes measured in non-fractionated serum correlates with that of the supernatant after p2p4 separation (Fig. 6c) but not with vesicular proteasomes (p2p4 fraction) (Fig. 6d), thereby suggesting that the variation of extracellular proteasome we detect in the CIS and RRMS patient sera is not due to the EVs’ content.

## Conclusions

Extracellular proteasome has been proposed as a suitable biomarker in some cancers and autoimmune diseases, potentially allowing for better diagnosis, patient stratification, and prediction of the response to therapy^18^,^19^,^20^,^21^, even though its function is not yet understood. Here, we elucidate one of its potential functions in the extracellular space. The OPN chemotactic activity toward three cell types is indeed enhanced by extracellular proteasome through the release of active OPN fragments during OPN processing. These peptide fragments are buried in the C-terminal portion of OPN-FL. When they are removed from the original protein they have likely lost their secondary conformation and the absence of surrounding bulky structures might facilitate an enhanced binding to chemotactic receptors. This could enhance their chemotactic activity towards migrating cells, such as HUVECs, PBLs and PBMs. Similar mechanism has been proposed for the activation of other cryptic sites of OPN-FL upon processing by thrombin^2^,^7^. The action of these novel OPN peptides seems to be sequence-specific since peptides with inverted sequences do not have chemotactic activity and small variations of the sequence alter the peptide efficacy. Indeed, the removal of a single residue from the peptide OPN_267-278_ remarkably reduces its chemotactic activity, whereas the removal of six residues from the peptide OPN_286-306_ confers a stronger chemotactic efficacy to the peptide (Fig. 3 and Supplementary Fig. 3). It is noteworthy that peptide hydrolysis might not be the only mechanism whereby extracellular proteasome modulates OPN activities. Indeed, proteasome is also able to ligate peptide fragments thereby generating novel peptides with sequences that are not present in the parental protein. This process, named proteasome-catalyzed peptide splicing, is much more frequent than expected^26^,^27^,^28^ and it has been already hypothesized to be involved in generating autoimmune epitopes in diseases such as MS^29^,^30^. Although proteasome-generated spliced peptides have been investigated so far only as target of T cell response, they might have other functions and cells might use them to multiply the variety of functional peptides derivable from a parental protein.

The cellular origins of the serum proteasome remain unknown, and we can only refer to the extensive study of Zoeger *et al*.^14^, who have shown how proteasome subtype profiles cannot be assigned to any of the investigated blood cells. Here, we demonstrate that although EVs contain proteasomes, only a minor portion of extracellular proteasome that we measure in sera is stored by EVs. This portion does correlate neither with total extracellular proteasome concentration nor with the variations we observe in CIS sera. We cannot exclude the possibility that extracellular proteasome is initially released in short-lived EVs, whose content is rapidly liberated into the extracellular space after the release from the cells. Indeed, the content of these short-lived EVs would be detected as free extracellular proteasome in our assays. However, multiple mechanisms of release are a common feature for both secretory molecules (ATP), or proteins lacking a conventional leader sequence (IL1-β or tau protein); these could be used also by proteasome.

It is noteworthy that, in this study, we investigate only the chemotactic effect of OPNs; however, OPNs also mediate other pro-inflammatory mechanisms involved in MS. Indeed, in EAE it has been demonstrated that OPN is able to trigger the neurological relapse by supporting the recruitment of autoimmune T cells into the CNS, by stimulating the expression of Th1 and Th17 cytokines, and by inhibiting T cell apoptosis through the regulation of Foxo3 and NF-kB transcription factors expression^6^. Additionally, activated T cells can secrete OPN, thus enhancing the Th1 and inhibiting the Th2 responses^2^. Recently, it has been demonstrated that the immunoproteasome is also involved in the regulation of Th1 and Th17 cytokine production and T cell differentiation^9^,^31^,^32^. Moreover, the administration of a selective immunoproteasome inhibitor prevents EAE progression and ameliorates a relapse when the treatment is started in the recovery phase. These effects depend on the reduction of immune cell infiltration into the brain and spinal cord, as well as the inhibition of Th17 cell differentiation^33^. Thus, based on our *in vitro* results, we might speculate that the role of immunoproteasome in promoting EAE could be, at least in part, the direct outcome of an altered activation of OPN pro-inflammatory mechanisms by extracellular immunoproteasome.

## Materials and Methods

### *In vitro* processing of cytokines by purified 20S proteasome

20S proteasomes are purified from human erythrocytes or T2 cell line, which contain only standard proteasome. The purity of 20S proteasome preparation has been previously shown^12^.

For the *in vitro* degradation assays, 0.4 μg recombinant OPN-FL (OPN_17-314_-6His), OPN-N (OPN_17-168_-6His) and OPN-C (OPN_169-314_-6His) are incubated in 20 μl solution at 37 °C with different concentration of erythrocyte 20S proteasome for 4 h (Fig. 1a), or 4 μg erythrocyte 20S proteasome for 20 h at 37 °C (Supplementary Fig. 2), or by 3.5 μg 20S proteasomes from the T2 cell line for 1–4 h (Fig. 1b). The ratio of OPNs/proteasomes used in the kinetics assays mimics that observed in serum of healthy controls and MS patients (Fig. 5), and that used in the cell migration assays (Fig. 2). Substrate degradation is detected by immunoblotting using antibodies for human OPN-N, or -C (1: 250 Maine Biotechnology Services), or OPN-FL (1: 400 Enzo Life science) as previously described^34^. A secondary anti-mouse or anti-rabbit horseradish peroxidase-conjugated antibody (1:5000; Calbiochem) is used for 1.5 h at room temperature followed by ECL detection (Amersham). The samples described in Supplementary Fig. 2 are analyzed by mass spectrometry (see below).

### OPN fragment identification and peptide synthesis

Liquid chromatography tandem mass spectrometry analyses are performed as previously described^35^ on a 4700 proteomics Analyzer (ABSCIEX, Framingham, MS) off-line coupled with a Dionex UltiMate 3000 RSLC system and Probot fractionation device (Thermo Scientific, Idstein, Germany). Mass spectrometry spectra are recorded in the range of *m*/*z* 600–4000 and with the accumulation of 1200 sub-spectra. Fragmentation spectra are measured from the five most intensive precursor ions (S/N > 40). 5000–10.000 laser shots are accumulated. The peak lists are generated by the “Peak to Mascot” tool of the 4000er Series Explorer v3.6. For data analysis the MASCOT server (version 2.3, Matrixscience, London, UK) is used. Database searches are performed using SwissProt (2014_08; 546238 protein sequences) and the following parameters: no enzyme, mass tolerances for precursor ions 100 ppm and for fragment ions 0.5 Da. Peptides of OPN-C produced by 20S proteasome *in vitro* are accepted as identified if they provide a MASCOT score for identity with p < 0.01. MALDI/TOF/TOF fragmentation spectrum of OPN-FL_217-230_ does not fulfill this criterion. However, its identity is verified by comparison with the fragment pattern of the synthetic analog. The reference peptide is synthesized in-house using Fmoc solid phase chemistry as previously described^35^, and have the following sequences: WDSRGKDSYETSQL (OPN_217-230_), KANDESNEHSDVIDSQEL (OPN_249-266_),SKVSREFHSHEF (OPN_267-278_), KVSREFHSHEF (OPN_268-278_), VVDPKSKEEDKHLKFRISHEL (OPN_286-306_), and KEEDKHLKFRISHEL (OPN_292-306_). Inverted peptides are designed by inverting the sequence of the related peptides identified in the proteasome-mediated digestion of the OPN-C have the following sequences: LQSTEYSDKGRSDW (_inverted_OPN_217-230_), LEQSDIVDSHENSEDNAK (_inverted_OPN_249-266_), FEHSHFERSVKS (_inverted_OPN_267-278_), and LEHSIRFKLHKDEEK (_inverted_OPN_292-306_).

### Spatial localization of OPN fragments within OPN-FL 3D structure

I-Tasser^36^ is used to predict the tertiary protein structure of OPN-FL. The c-score (value between -5 and 2, where higher c-scores indicate higher confidence in the predicted structure) of the best model predicted by I-TASSER is −3.33, which is comparable to the confidence obtained by predicting the structure of OPN-C^37^. Pymol is used for graphic visualization (The PyMOL Molecular Graphics System, Version 1.7.4 Schrödinger, LLC).

### Cell culture

HUVECs are isolated from human umbilical veins via trypsin treatment (1%) and cultured in M199 medium (Sigma-Aldrich) with the addition of 20% FCS (Invitrogen, Burlington, ON, Canada) and 100 U/mlpenicillin, 100 mg/ml streptomycin (Invitrogen), 5 UI/ml heparin (Sigma-Aldrich), 12 mg/ml bovine brain extract, and 200 mM glutamine (Hyclone Laboratories). HUVECs are grown to confluence in flasks and used at the second to fifth passage. The use of HUVECs is approved by the Ethics Committee of the “Presidio Ospedaliero Martini” of Torino and conducted in accordance with the Declaration of Helsinki. Written informed consent is obtained from all donors. 2*10^3^ HUVEC are used for migration assay and cultured in M200 medium (GIBCO) with 100 U/ml penicillin, 100 mg/ml streptomycin and 200 mM glutamine.

Peripheral blood mononuclear cells are separated from buffy coat, provided by the local Blood Transfusion Service of Novara, Italy, with the Ficoll-Hypaque reagent (Limpholyte-H, Cedarlane Laboratories) by density-gradient centrifugation. PBLs are obtained from peripheral blood mononuclear cells cultured in RPMI 1640 (Euroclone) supplemented with 10% FBS, L-glutamine, penicillin-streptomycin, and let 2 h on plate to remove the adherent monocytes; PBMs (CD14^+^) are isolated with the EasySep^TM^ Human CD14 Negative Selection Kit (StemCells Techologies, Vancouver, BC, USA). For both cell types the cell purity is checked by immunophenotypic analysis and is higher than 98%. 5*10^4^ PBLs or 2*10^4^ PBMs are used for migration assay and cultured in medium X-vivo 20 or X-vivo 15 (Lonza), respectively, with 100 U/ml penicillin, 100 mg/ml streptomycin and 200 mM glutamine.

### Cell migration assay

In the Boyden chamber (BD Biosciences) migration assay, cells are plated onto the apical side of 50 μg/ml Matrigel-coated filters (0.5 μm pore size, Neuro Probe, BIOMAP snc) in M200 serum-free medium for HUVECs or X-vivo serum-free medium for PBLs or PBMs.

10 μg/ml OPN-FL, OPN-C and OPN-N (Fig. 2) or 10–500 nM OPN-C peptides (Fig. 3 and Supplementary Fig. 3), are placed in the basolateral chamber; 10 ng/ml vascular endothelial growth factor-α (VEGF-α; Sigma-Aldrich) or 1 ng/ml recombinant human RANTES (rh RANTES/CCL5; ImmunoTools GmbH) are placed as reference chemoattractant for the HUVECs or the PBLs and the PBMs, respectively.

20S proteasome is pre-incubated with OPN-FL, OPN-C and OPN-N (with ratio ng proteasome: ng OPNs = 20:1) for 2 h at 37 °C under 5% CO_2_ and subsequently placed in the basolateral chamber.

The chamber is incubated at 37 °C (5% CO_2_). After 20 h, the cells on the apical side are wiped off with Q-tips. The cells on the bottom of the filter are stained with crystal violet (HUVECs), or eosin Y and thiazine (PBLs and PBMs), and all counted with an inverted microscope (magnification x40). Data are shown as percentage of the treated cells migration *vs* the control migration measured for untreated cells. Control migration of the experiments shown in Fig. 2 is (mean ± SEM) 287 ± 39 cells for HUVECs (n = 38), 175 ± 28 for PBLs (n = 18), and 250 ± 32 for PBLs (n = 13).

### Proteasome release in cell cultures

7*10^4^ HUVECs are seeded in 48 well plates with M199 20% FCS medium. After 20 h HUVECs are twice washed with M200 medium and refilled with M200 medium. Then, HUVECs are treated with 10 μg/ml OPN-FL, OPN-C, OPN-N or 10 ng/ml VEGF-α. After 6 h culture the supernatant is collected. Four replicate wells are used for each donor (n = 14).

1 × 10^5^ lymphocytes are seeded in 96 well plates with RPMI 1640 medium + supplements. After 20 h lymphocytes are washed and refilled with X-Vivo 20 medium. Then, lymphocytes are treated with 10 μg/ml OPN-FL, OPN-C, OPN-N or 1 ng/ml RANTES. After 20 h culture the supernatant is collected. Four replicate wells are used for each donor (n = 10).

Medium supernatant is processed by ELISA assay to measure the proteasome concentration (see below).

Trypan blu assay is assessed in order to evaluate cell viability. Viable cells are measured, by 2,3-bis(2-methoxy-4-nitro-5sulphophenyl)-2H- tetrazolium-5-carboxanilide (Sigma-Aldrich) inner salt reagent, at UV 570 nm, as described by the manufacturer’s protocol.

### Donor enrolment, blood drawing, serum separation

The blood samples of the Italian cohorts are obtained after informed consent and ethical approval by the University of Piemonte Orientale “Amedeo Avogadro” (Novara; ethical committee approval: CE 18/04), IRCCS Fondazione Ospedale Maggiore Policlinico and Ospedale San Raffaele (Milano; ethical committee approval: Banca-INSPE and MSGENE02). The blood samples of the German cohort are obtained after informed consent and ethical approval by the Clinical and Experimental MS Research Centre, Charité – Universitätsmedizin Berlin (Berlin; ethical committee approval: EA1/182/10). All methods and procedures are performed in accordance with the relevant guidelines and regulations of the ethical committee approvals. RRMS and CIS patients as well as age-, gender-, and ethnicity-matched healthy controls without a history of any neurological or other chronic disease are recruited (Supplementary Table 1). All participants are older than 18 years; an information sheet related to the study is provided and an informed consent is undersigned by each participant. Diagnosis of MS is made according to the McDonald 2010 criteria^38^. The clinical workup and examinations of MS patients at the visit include: detailed medical and demographic history, neurological examination, determination of the expanded disability status scale score, brain Magnetic Resonance Imaging, type of therapy (Glatiramer acetate or IFN-1β). Inclusion criteria are the following: age > 18 years, a first clinical event suggestive of central nervous system demyelination within 6 months before inclusion into the study for CIS patients or a diagnosis of RRMS according to the McDonald 2010 criteria^38^ within 24 months before inclusion into the study. Exclusion criteria are the inability or unwillingness to provide informed consent, a history of alcohol or drug abuse, any ocular diseases precluding performance of optical coherence tomography, and any conditions (*e.g.* allergies) or devices (*e.g*. cardiac pacemaker) precluding MRI examinations. Serum samples of RRMS patients are obtained from 10 ml of peripheral blood, which are drawn either at the time of relapse (2–7 days from its onset) or in remission (after 1–12 months from the last relapse) as previously reported^39^. The RRMS patients receive a corticosteroid treatment after the blood withdrawal during the relapse.

### Extracellular proteasome and OPN quantification in serum

The concentration of the extracellular proteasomes is measured in serum by enzyme-linked immunoabsorbent assay (ELISA), as previously described with minor modifications^40^. Plates (FA9439454, Nunc Immuno MaxiSorp Surfaces, Thermo Fisher Scientific Inc.) are coated overnight with mouse monoclonal antibodies towards 20S proteasome subunit α6 (PW 8100, Enzo Life Sciences Inc.) 1:1500 in 100 μL PBS at pH 7.4. The plates are washed 5 times in PBS-Tween20 (PBST) 0.1% and incubated 6 h at room temperature with a blocking solution of PBST-BSA 1% to prevent non-specific bindings of the samples. Samples are diluted in PBST-BSA 1% (serum 1:15–1:30; plasma/serum supernatant 1:15; medium of *in vitro* experiments 1:2.5–1:10) and 100 μl are applied to each well overnight at 4 °C. Standard curves are established for every plate using purified 20S proteasomes with concentrations ranging from 0 to 100 ng/ml (8 linear dilution steps). The plates are washed 3 times and 100 μl of anti-proteasome rabbit polyclonal antibody 1:750 (K42, in house) in PBST solution are added for 3 h at 4 °C. After additional washing steps, 100 μl of 1:15000 peroxidase-conjugated mouse anti-rabbit IgG (11-035-003, Jackson ImmunoResearch Laboratories Inc.) in PBST solution are used for antigen detection (1 h at 4 °C). Finally, plates are washed five times and 100 μl of TMB substrate added (T0440, Sigma-Aldrich). The reaction is stopped with sulphuric acid and OD-values are determined at 450 nm. Serum from a young healthy control is added in each plate as internal control.

Serum OPN concentration is evaluated by ELISA according to the protocol provided by the manufacturer (Calbiochem) as previously described^39^. The optical density is measured with a microplate reader (Bio-Rad).

All assays are performed in duplicate in three independent measurements, and the observer is blinded to the diagnosis.

### Analysis of EVs

100–200 μl of human serum and plasma are diluted 1:5 in PBS and processed by serial centrifugations to collect EVs^41^ into p2p3, p4 or p2p4 fractions. The supernatants are recovered and analyzed for extracellular proteasome content by ELISA, as above described, thereby reckoning the proportion of extracellular proteasomes in the supernatant *vs* unfractionated sample. The pellets are lysed in 20 μl of RIPA buffer supplemented with Protease Inhibitor Cocktail (P2714, Sigma Aldrich) and the protein content is analyzed by SDS-PAGE and western blotting, as previously described^42^. Anti-α6 proteasome subunit antibody (PW 8100, Enzo Life Sciences Inc.) is used to measure total proteasomes content within isolated EVs. Crude protein extract from lymphoblastoid cell line is used as control and for normalizing data of samples loaded in different gels. Equal loading of EVs proteins is assessed by SDS-PAGE and Coomassie Blue gel staining.

### Statistical analysis

For the statistical analysis, peripheral blood samples are analyzed separately in the following categories: RRMS in relapse, RRMS in remission and healthy controls for Italian samples, and CIS, RRMS in remission and healthy control cohorts for German samples. For each sample, serum levels of extracellular proteasome and OPN contents are measured in triplicate and the mean value is calculated. Each cohort is tested to assess whether extracellular proteasome correlated to gender or age of the donors, as well as to the clinical history and parameters measured at the withdrawal (data not shown). Data are tested for normality distribution and homoscedasticity by Shapiro-Francia, Shapiro-Wilk and Skewness-Kurtosis tests. To identify significant difference between groups, One-way ANOVA, Mann-Whitney or Kruskal-Wallis tests are applied depending on the underlying distributions. To identify significant variation in proteasome release or chemotaxis in cell culture upon different stimuli we apply Wilcoxon test for paired samples. Pearson’s correlation coefficients are computed for correlation analyses. Descriptive statistics are carried out with STATA v.9.0 (Stata Corp.), SPSS (version 17) and R; a *p*-value < 0.05 is considered statistically significant. After applying Bonferroni correction method for multiple comparisons, the *p* value threshold for significance is set at p = 0.0083. A bootstrap test (1000 samples using 100% of the data) is performed to obtain confidence intervals for the correlation coefficient between OPN and 1/extracellular proteasome. We extract the bootstrap distribution of the correlation coefficient as well as the 5% and 95% quantiles for the confidence intervals.

## Additional Information

**How to cite this article**: Dianzani, C. *et al*. Extracellular proteasome-osteopontin circuit regulates cell migration with implications in multiple sclerosis. *Sci. Rep.* **7**, 43718; doi: 10.1038/srep43718 (2017).

**Publisher's note:** Springer Nature remains neutral with regard to jurisdictional claims in published maps and institutional affiliations.

## Supplementary Material

Supplementary Information
