# Supplementary material for: Extracellular proteasome-osteopontin circuit regulates cell migration with implications in multiple sclerosis
Source: Sci Rep. 2017 Mar 9;7:43718. doi: 10.1038/srep43718 (PMC5343429; doi:10.1038/srep43718)
Supplement: Supplementary Information [file srep43718-s1.pdf]

Extracellular proteasome-osteopontin circuit regulates cell migration with implications in multiple sclerosis

Chiara Dianzani[1,#], Elena Bellavista[2,#], Juliane Liepe[3], Claudia Verderio[4,5], Morena Martucci[2], Aurelia Santoro[2], Annalisa Chiocchetti[6], Casimiro Luca Gigliotti[6], Elena Boggio[6], Benedetta Ferrara[1], Loredana Riganti[4], Christin Keller[7,8], Katharina Janek[7], Agathe Niewienda[7], Chiara Fenoglio[9], Melissa Sorosina[10], Roberto Cantello[11], Peter M. Kloetzel[7,8], Michael P.H. Stumpf[3], Friedemann Paul[12,13,14], Klemens Ruprecht[13,15], Daniela Galimberti[9], Filippo Martinelli Boneschi[10], Cristoforo Comi[6,11], Umberto Dianzani[6] & Michele Mishto[7,8,16,$]

1 Department of Drug Science and Technology, University of Turin, 10126 Torino, Italy.

2 Department of Experimental, Diagnostic and Specialty Medicine (DIMES), University of Bologna, 40126 Bologna, Italy.

3 Centre for Integrative Systems Biology and Bioinformatics, Department of Life Sciences, Imperial College London, SW7 2AZ London, UK.

4 Institute of Neuroscience, Centro Nazionale delle Ricerche (CNR), 20129 Milano, Italy.

5 IRCCS Humanitas, 20089 Rozzano, Italy.

6 Interdisciplinary Research Centre of Autoimmune Diseases (IRCAD), University of Piemonte Orientale, Amedeo Avogadro, 28100 Novara, Italy.

7 Institut für Biochemie, Charité - Universitätsmedizin Berlin, 10117 Berlin, Germany.

8 Berlin Institute of Health, 10117 Berlin, Germany.

9 Neurology Unit, Department of Pathophysiology and Transplantation, University of Milan, "Dino Ferrari" Centre, Fondazione IRCCS Cà Granda, Ospedale Maggiore Policlinico, 20100 Milano, Italy.

10 Department of Neuro-rehabilitation and Laboratory of genetics of Neurological complex disorders, Institute of Experimental Neurology (INSPE) Scientific Institute San Raffaele, 20132 Milano, Italy.

11 Department of Translational Medicine, Section of Neurology, University of Piemonte Orientale, 28100 Novara, Italy

12 AG Klinische Neuroimmunologie des Neurocure Clinical Research Center, Charité - Universitätsmedizin Berlin, 10117 Berlin, Germany.

13 Clinical and Experimental Multiple Sclerosis Research Centre - Universitätsmedizin Berlin, 10117 Berlin, Germany.

14 Experimental and Clinical Research Center, Max Delbrueck Center for Molecular Medicine and Charité - Universitaetsmedizin Berlin, Berlin, Germany

15 Department of Neurology, Charité - Universitätsmedizin Berlin, 10117 Berlin, Germany.

16 Department of Health Science, University of Piemonte Orientale, 28100 Novara, Italy

[#] Elena Bellavista and Chiara Dianzani equally contributed to the work.

[$] To whom the correspondence should be addressed: Michele Mishto, Institut für Biochemie, Charité - Universitätsmedizin Berlin, Charitéplatz 1, 10117 Berlin, Germany. Tel: +49(0)30450528-288/071. Email: michele.mishto@charite.de.

List of Supplementary material

Supplementary table 1	Characteristics of healthy control and patient cohorts
Supplementary figure 1	Extracellular 20S proteasome breaks OPN molecules
Supplementary figure 2	Tandem mass spectrometry identification of the six studied fragments of OPN-C generated during *in vitro* digestion by 20S proteasomes
Supplementary figure 3	Dose-dependent chemotactic effect of proteasome-generated OPN fragments

		**Healthy Controls**	**RRMS Rem**	**RRMS Rel**	**CIS**
**Italian population**	Age (y)	36.9 ± 14.8	42.3 ± 10.3	36 ± 10.4	
	Count (n)	62	50	25	
	Gender (M/F)	17/45	22/28	9/16	
	Disease onset (y)	-	41.4 ± 11.2	31.8 ± 9.4	
	Disease duration (y)	-	9.7 ± 7.4	5.0 ± 5.7	
	MSSS	-	3.0 ± 2.2	4.9 ± 2.5	
**German population**	Age (y)	27.2 ± 4.0	36.8 ± 11.9		32.8 ± 8.8
	Count (n)	50	13		37
	Gender (M/F)	23/27	5/8		15/22
	Disease onset (y)	-	33.1 ± 11.6		31.3 ± 8.8
	Disease duration (y)	-	2.3 ± 1.0		1.2 ± 0.6
	MSSS	-	4.7 ± 2.8		-

Supplementary table 1. Characteristics of healthy control and patient cohorts. Continuous variables are reported as mean ± SD. M = male, F = female, MSSS = Multiple Sclerosis Severity Scale.

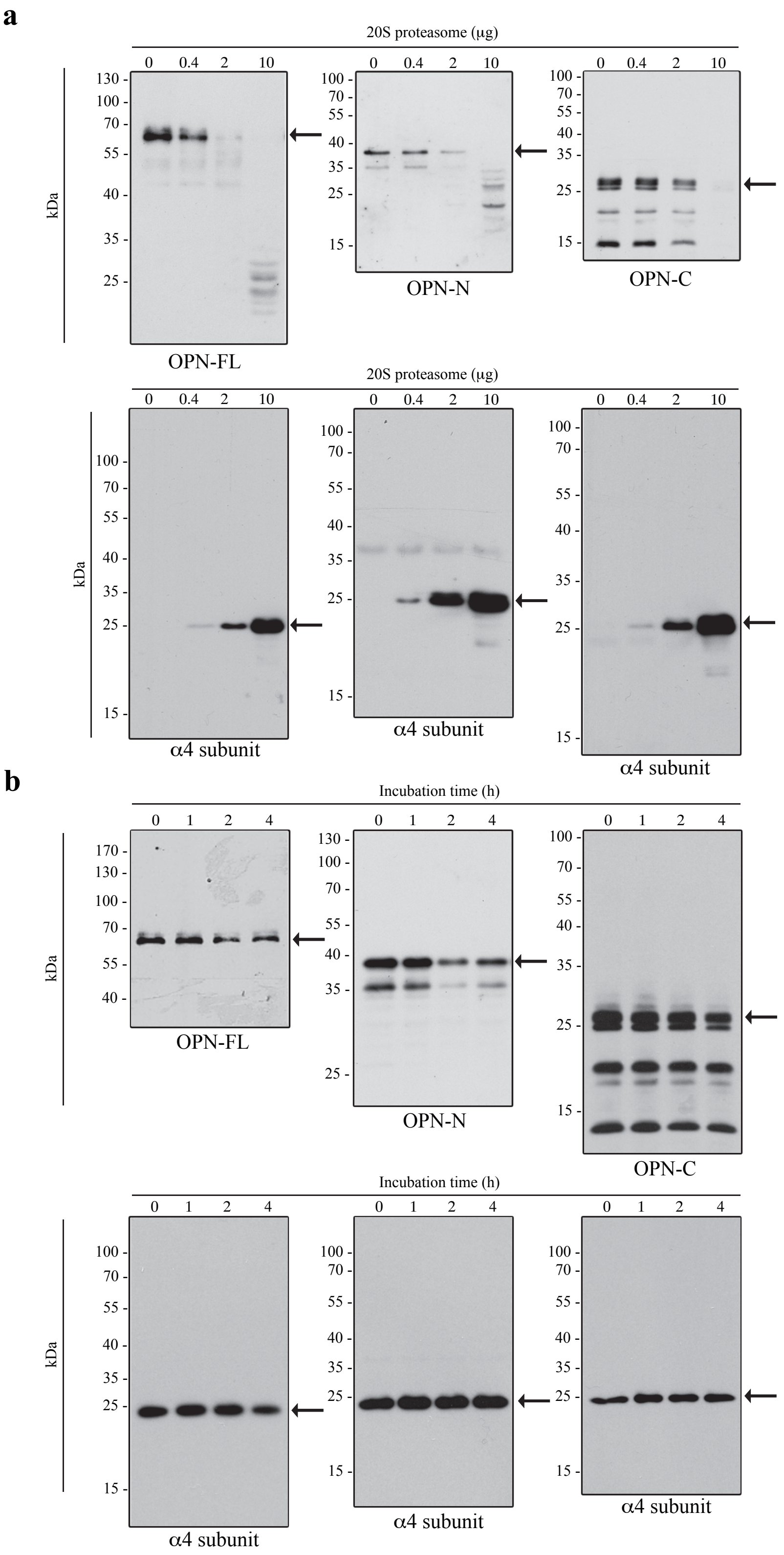

Supplementary figure 1. Extracellular 20S proteasome breaks OPN molecules. (**a**) Representative *in vitro* digestion (n = 3) of recombinant OPN-FL, OPN-N, OPN-C by different amount of human erythrocyte 20S standard proteasome. The band pattern visible in the samples with 10 μg proteasome resembles that of the proteasome subunits and is thus likely the outcome of a cross-reactivity of the antibodies with such a large amount of proteasome. (**b**) Degradation kinetics of OPN-FL, OPN-N and OPN-C by T2 20S standard proteasomes are shown by representative Western Blot assay of 4-5 independent experiments. (**a-b**) The full Western blots are shown. The relevant bands of the OPNs (upper panels) or the proteasome α4 subunits (as controls; lower panels) are marked with an arrow and they are shown in Fig. 1.

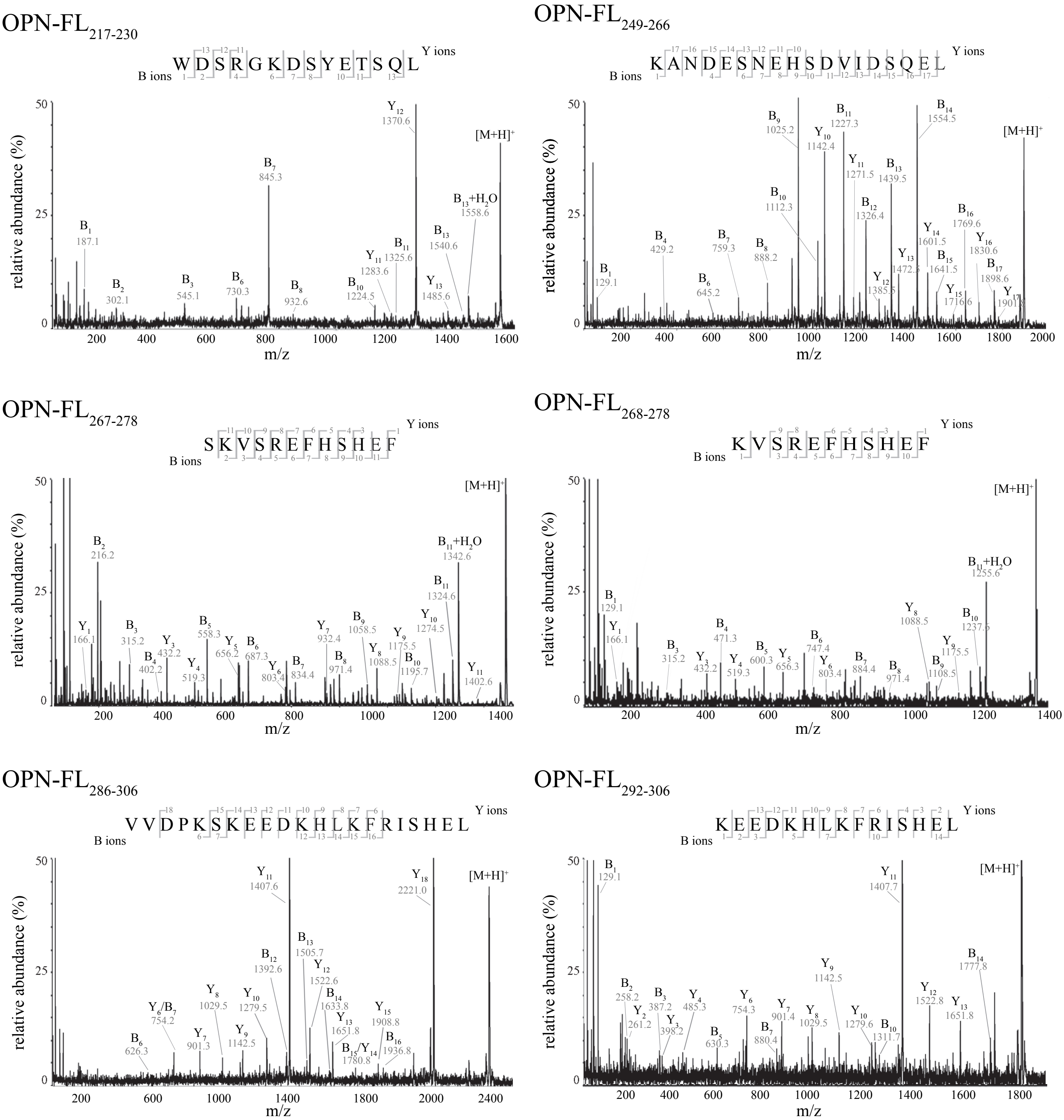

Supplementary figure 2. Tandem mass spectrometry identification of the six studied fragments of OPN-C generated during *in vitro* digestion by 20S proteasomes. MALDI-tandem mass spectrometry spectra of the peptides WDSRGKDSY-ETSQL ($OPN_{217-230}$) [peptide score = 59, p = 0.097], KANDESNEHSDVIDSQEL ($OPN_{249-266}$) [peptide score = 101, p = 5.8*10-6], SKVSREFHSHEF ($OPN_{267-278}$) [peptide score = 96, p = 2.1*10-5], KVSREFHSHEF ($OPN_{268-278}$) [peptide score = 72, p = 0.0057], VVDPKSKEEDKHLKFRISHEL ($OPN_{286-306}$) [peptide score = 75, p = 0.0027], and KEEDKHLKFRISHEL ($OPN_{292-306}$) [peptide score = 69, p = 0.0099] identified in the *in vitro* digestion of the substrate OPN-C by erythrocyte 20S proteasome for 20 h. The identity of the peptide $OPN_{217-230}$ is verified by comparison with the fragment pattern of the synthetic analog.

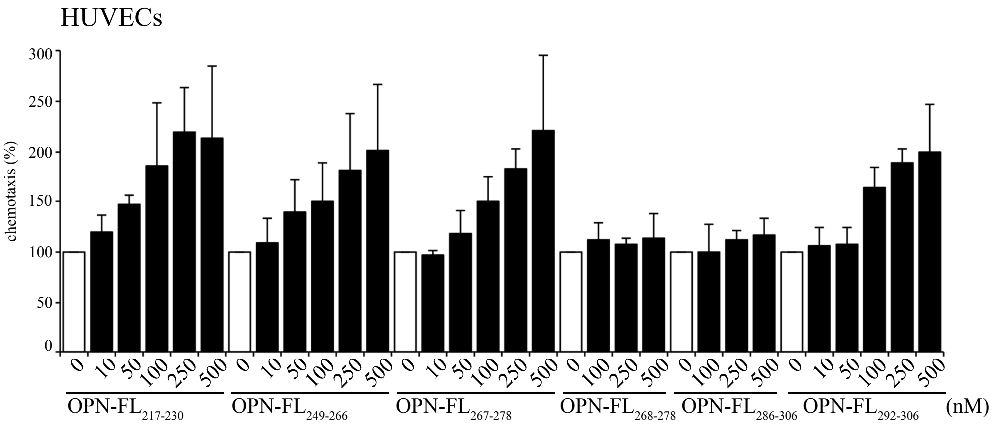

Supplementary figure 3. Dose-dependent chemotactic effect of proteasome-generated OPN fragments. Effect of different concentrations of OPN fragments (10 - 500 nM) on the HUVEC chemotaxis is depicted. Values are reported as percentage of treated vs untreated cells that migrated after 20 h and they are expressed as the mean and the SD of independent experiments (n = 5 - 14). Cell migration is measured in the Boyden chamber migration assay.
